# Role of microRNA-33a in regulating the expression of PD-1 in lung adenocarcinoma

**DOI:** 10.1186/s12935-017-0474-y

**Published:** 2017-11-17

**Authors:** Laura Boldrini, Mirella Giordano, Cristina Niccoli, Franca Melfi, Marco Lucchi, Alfredo Mussi, Gabriella Fontanini

**Affiliations:** Department of Surgical, Medical, Molecular Pathology and Critical Area, University of Pisa, Via Roma 57, 56126 Pisa, Italy

**Keywords:** miR-33a, PD-1, Lung adenocarcinoma

## Abstract

**Background:**

MiRNAs are vital in functioning as either oncogenes or tumor suppressors in the cell cycle. Target transcripts for immune checkpoint molecules such as PD-1/PD-L1 and (programmed cell death-1/its ligand and cytotoxic T-lymphocyte antigen 4) have proven to be beneficial against several solid tumors, including lung adenocarcinoma.

**Methods:**

Simultaneous quantification of the expression level of miR-33a and *PD*-*1*, *PD*-*L1* and *CTLA4* mRNAs with NanoString technology was performed in 88 lung adenocarcinoma specimens. A cohort of 323 lung adenocarcinoma patients from the cancer genome atlas (TCGA) database was further analyzed, in order to test our hypothesis. Potential interference of *PD*-*1, PD*-*L1* and *CTLA4* gene expression by miR-33a was predicted using the microRNA target prediction program *RNA22.*

**Results:**

High miR-33a expression was significantly associated with younger (p = 0.005), female (p = 0.04), patients with low grade (p < 0.0001), early stage (p = 0.03) tumors, and better survival. The hypothesis of the involvement of miR-33a in PD-1/PD-L1/CTLA4 mechanisms was corroborated by the finding of putative miR-33a binding sites in all three genes using the *RNA22* method. We found an inverse correlation between miR-33a and *PD*-*1* levels (p = 0.01), as well as for *PD*-*L1* (p = 0.01) and *CTLA4* (p = 0.03) expression, and a significant better prognosis for patients with high miR-33a/low *PD*-*1*. TCGA database analysis confirmed that miR-33a high levels were associated with low PD-1 expression and with longer survival on a larger population.

**Conclusions:**

Our study emphasizes the notion of a potential value of miR-33a as a favorable prognostic marker through *PD*-*1* regulation.

## Background

MicroRNAs (miRNAs) are small non-coding RNA molecules that function as indispensable regulators of an increasing number of cellular processes [1–3]. miRNAs are vital in regulating cell proliferation and apoptosis, in addition to functioning as either oncogenes or tumor suppressors in the cell cycle [4]. In a recent study [5], miR–33a was identified as having potential tumor–suppressive activity. Overexpression of miR–33a was demonstrated to inhibit the growth of lung cancer cells, but the exact role of individual miRNAs strictly depends on their expression pattern and their targeted genes. Lung cancer is the most common cancer worldwide and despite recent progress with molecularly targeted agents, its prognosis is usually poor and new strategies need to be developed. Cancer immunotherapy is emerging as a very promising therapeutic strategy and recently various clinical trials exploring the use of anti-programmed cell death protein 1 (PD-1, also known as CD279), PD-L1 (programmed cell death-1 ligand, also known as CD274) and CTLA4 (cytotoxic T-lymphocyte antigen 4) inhibitors have successfully shown antitumor activities in lung cancer [6–8]. For most cancer biomarkers, determination of the levels of such immune function markers in formalin-fixed, paraffin-embedded (FFPE) samples has been generally performed by conventional immunohistochemistry using various antibodies with various levels of validation. Different thresholds have been used to define PD-1/PDL1 positivity, but cut-off points as the proportion of membrane-positive tumor cells were subjective in terms of estimated visual levels, such that reproducibility has not been formally assessed in the clinical setting.

Moreover, there are limited data on the prognostic significance of PD-1/PD-L1, with some showing poor [9], better prognosis [10] or no prognostic role [11]. Another approach to regulating the immune response in the tumor microenvironment is by modulating the level of miRNAs. A recent review [12] emphasized the role of miRNAs as modulators of immune checkpoint molecules and consequently as potential cancer therapeutic targets.

Here, we investigated the potential role of miR-33a and demonstrated its potential value as prognostic marker by regulating *PD*-*1/PD*-*L1* and *CTLA4* expression in lung adenocarcinoma.

## Materials and methods

### Patients

Eighty-eight lung adenocarcinoma patients were retrospectively selected among those operated at the Unit of Thoracic Surgery of the A.O.U.P. Histological diagnoses were independently formulated according to the World Health Organization classification [13–15]. Clinical–pathological characteristics were collected whenever available for all the patients.

### RNA isolation

Total RNA were isolated from a representative area selected and marked on the surface of 10 micron sections of formalin-fixed, paraffin-embedded (FFPE) tissues using the miRNeasy FFPE Kit (Qiagen Inc., Hilden, Germany) according to the manufacturer’s instructions. The quality and concentration of RNA was assessed using a NanoDrop spectrophotometer (Thermo-Scientific, Wilmington, Del).

### NanoString nCounter^**®**^ assay, data normalization and analysis

Expression of the selected miR-33a, *PD*-*1, PD*-*L1* and *CTLA4* genes among a miRGE panel was measured using the NanoString nCounter technology (NanoString Technologies, Seattle, WA). The nCounter measures total counts of mRNAs/miRNA by a multiplex hybridization assay followed by scanning and digital readouts of fluorescent probes [16]. Raw NanoString counts for each gene were subjected to a technical normalization, considering the counts obtained for positive control probe sets, followed by a biological normalization, using three reference genes included in the CodeSet, according to NanoString Technologies’ guidelines. The nCounter custom code set used in this study included three genes (PD-1, PD-L1 and CTLA4) with five housekeeping genes for reference [clathrin heavy chain 1 (CLTC), beta-glucuronidase (GUSB), tubulin beta (TUBB), hypoxanthine phosphoribosyltransferase (HPRT1), phosphoglycerate kinase 1 (PGK1)]. MiR-33a expression was tested simultaneously with other selected miRNAs and the normalization was performed using a scaling factor based on miRNAs with the lower variability coefficients.

*The Cancer Genome Atlas* (*TCGA) database.* From the TCGA data portal (http://tcga.cancer.gov/; accessed October 2017), we extracted PD-1 and miR-33a expression together with the corresponding clinical–pathological characteristics and survival data for 323 adenocarcinoma patients (LUAD).

### Target prediction

Potential regulation of *PD*-*1, PD*-*L1* and CTLA4 gene expression by miR-33a was predicted using the microRNA target prediction program *RNA22*, a pattern-based approach for the discovery of microRNA binding sites and their corresponding microRNA/mRNA complexes [17].

### Statistical analysis

Once the RNA hybridization data had been correctly prepared, the data were subjected to 2-way hierarchical clustering analysis (HCA) using nSolver 2.5 Analysis Software. Differential expression was determined by applying the non-parametric Wilcoxon test in order to determine the association between miR-33a expression and the clinical–pathological parameters. Survival analyses were performed using the Kaplan–Meier method with log-rank test and the Cox proportional hazard model. Statistical analyses were performed using JMP10 software (SAS, Milan, Italy), and a two-tailed p value < 0.05 was considered significant.

## Results

### Patient characteristics

This study included 88 patients with lung adenocarcinoma, 56 males and 32 females, with an age at diagnosis ranging from 30 to 81 years (mean 58.9 years, median 54.5). The predominant histologic patterns were characterized as follows: lepidic (29/88, 33%), solid (26/88, 29.5%), acinar (22/88, 25%), and papillar (11/88, 12.5%) variants. According to the degree of differentiation, three tumors were G1, whereas 58 and 27 were G2 and G3, respectively. The adenocarcinomas were all invasive. Their stages were classified as IA (17), IB (23), IIA (13), IIB (9), IIIA (23), IIIB (1), and IV (2). The survival data, with disease-free interval (DFI) and overall survival (OS), were available for all the patients and was last updated in March 2015. Concerning the smoking habits, there were 17 non-smokers, 16 former smokers, and 23 current smokers; for 33 patients, smoking history was unknown.

### miR-33a expression and clinical–pathological characteristics

miR-33a expression was compared with the patients’ clinical–pathological characteristics. High miR-33a expression was significantly associated with younger (p = 0.005), female (p = 0.04) patients with low grade (p < 0.0001), early stage (p = 0.03) tumors (Table 1).

**Table 1:** miR-33a expression in 88 lung adenocarcinoma patients

Variables (n)	miR33a expression (mean ± SD)	p
Age		
Young (44)	276.4 ± 24.9	
Old (44)	175.2 ± 24.8	*0.005*
Gender		
Male (56)	197.8 ± 22.6	
Female (32)	274.8 ± 29.9	*0.04*
Adenocarcinoma prevalent pattern		
Lepidic (29)	242.7 ± 31.8	
Solid (26)	185.3 ± 33.6	0.25
Acinar (22)	272.9 ± 36.5	
Papillar (11)	182.6 ± 51.6	
Tumor grading		
G1 (3)	634 ± 88.5	
G2 (58)	231.3 ± 20.1	
G3 (27)	168.6 ± 29.5	*<* *0.0001*
Stage		
I (40)	267.1 ± 26.7	
II–III–IV (48)	191.3 ± 24.4	*0.03*

### miR-33a and survival analysis

**Fig. 1:**
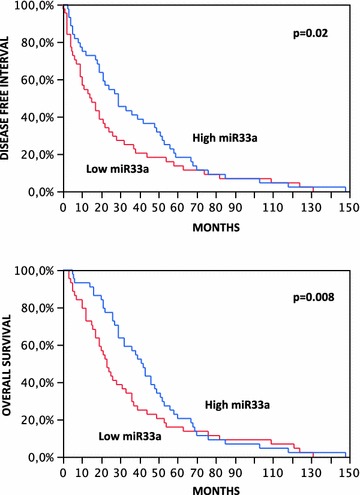
Kaplan-Meier survival analysis [disease-free interval (DFI) in the upper panel and overall survival (OS) in the lower panel] of 88 lung adenocarcinoma patients with distinct *miR*-*33a* expression

A Kaplan–Meier survival analysis using DFI (range 0–148, median 21 months) and OS (range 3–148, median 31.5 months) as endpoints and miR-33a expression as a dichotomous variable, distinguishing tumors with low from tumors with high expression, showed a significant better prognosis in patients with high miR-33a levels (Wilcoxon test, p = 0.02 and p = 0.008, for DFI and OS, respectively) compared to those with low levels (Fig. 1).

### miR-33a target prediction

**Fig. 2:**
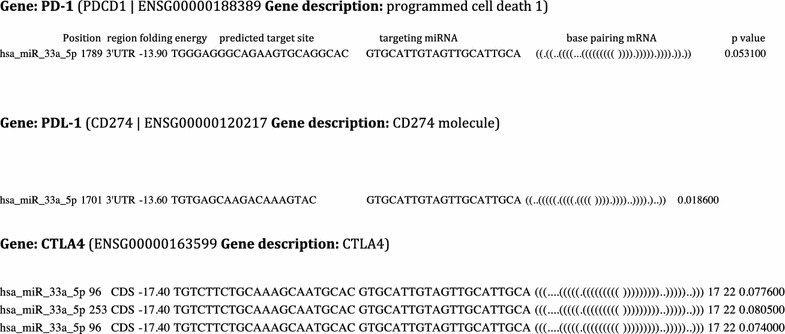
Prediction of alignment of miRNAs with *PD*-*1 (PDCD1), PD*-*L1 (CD274)* and *CTLA4* mRNAs based on the *RNA22* target prediction program analysis

To investigate the putative involvement of miR-33a in the regulation of the most studied immune checkpoint molecules (*PD*-*1, PD*-*L1,* and *CTLA4*), we used *RNA22* software for the discovery of microRNA binding sites and their corresponding microRNA/mRNA complexes. The *RNA22* method suggests that the number of microRNA binding sites may be greater than hypothesized and that microRNA regulation may be effected through the 5′UTR and CDS of gene transcripts in animals, in addition to 3′UTRs. Specifically, the hypothesis of the involvement of miR-33a in PD-1/PD-L1/CTLA4 mechanisms was corroborated by the observation that the 3′ untranslated region (UTR) of the *PD*-*1* mRNA carries a putative miR-33a binding site at position 1789. Two miR-33a binding sites for *CTLA4*, at positions 96 and 253 of the CDS region, and one in the 3′UTR of *PD*-*L1* (position 1701) were also found (Fig. 2).

### Correlation between miR-33a and PD-1 expression

We evaluated the abundance of PD-1, as well as of PDL-1 and CTLA4, mRNA by NanoString technology. The samples were divided into high and low expression groups based on the median fold-change value (1.76-fold change value ± 7.34 for PD-1; 2.79 ± 11.5 for PDL-1, and 30.23 ± 46.08 for CTLA4). Samples with low PD-1 mRNA levels also demonstrated low protein expression, assessed in FFPE tissue samples by immunohistochemistry using the anti-PD-1 mouse monoclonal antibody NAT105 (Ventana Medical Systems, Inc. USA) (data not shown).

**Fig. 3:**
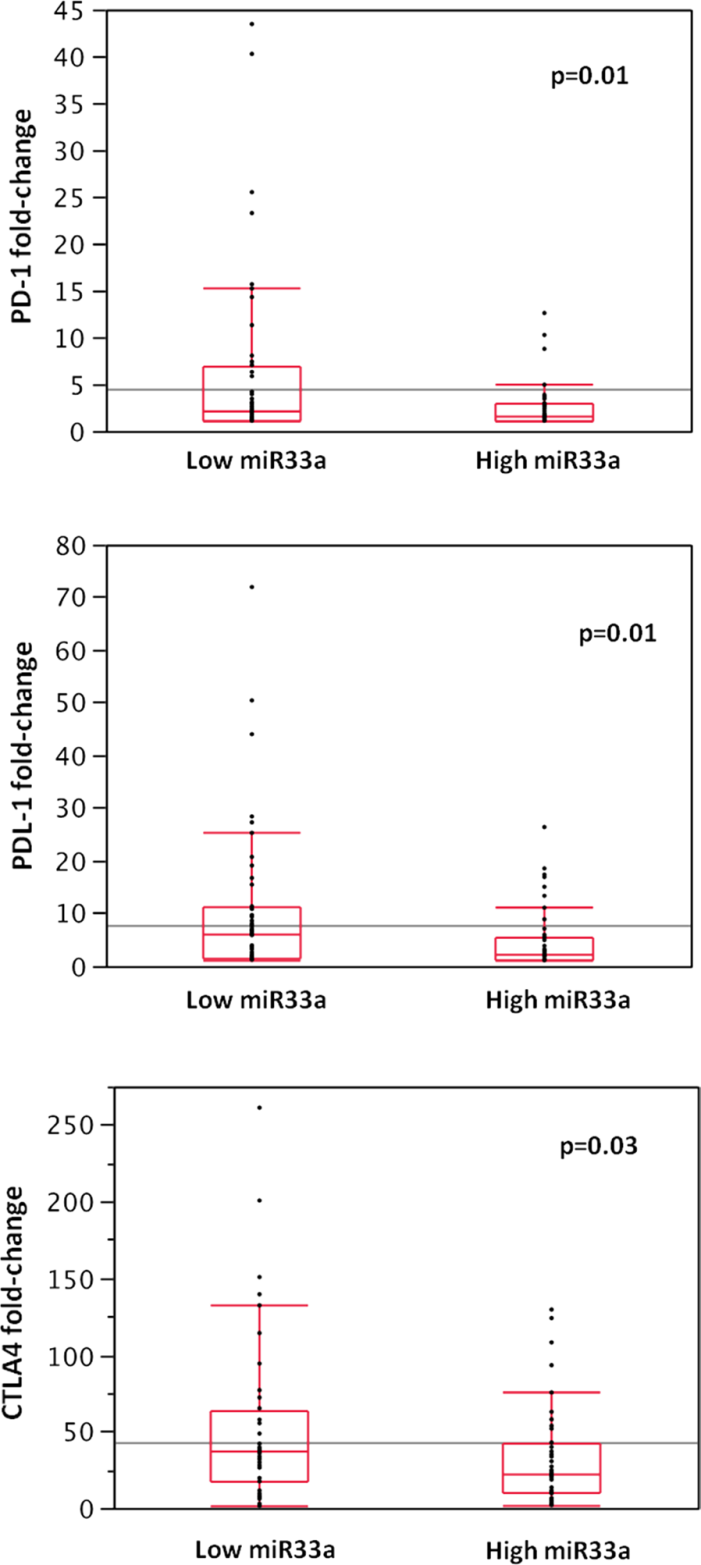
Downregulation of *PD*-*1, PD*-*L1* and *CTLA4* mRNA by miR-33a. Error bars on the bar charts represent the standard deviations

**Fig. 4:**
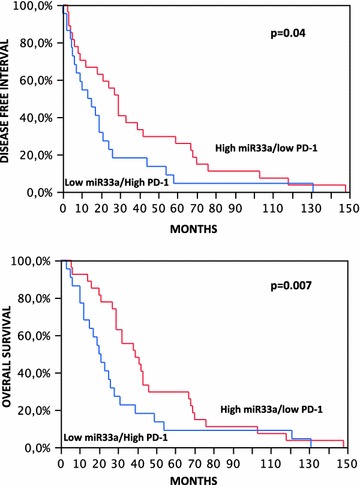
Kaplan-Meier survival analysis [disease-free interval (DFI) in the upper panel and overall survival (OS) in the lower panel] of 88 lung adenocarcinoma patients stratified according to *miR*-*33a* and *PD*-*1* co-expression. Patients with high miR-33a/lowPD-1 tumors had better survival rates than patients with low miR-33a/lowPD-1 tumors

We then analyzed their relationship with the expression level of miR-33a and found a statistically significant inverse correlation between miR-33a and *PD*-*1/PD*-*L1* expression. MiR-33a levels were lower in patients with higher *PD*-*1* (Chi square test, p = 0.01), *PD*-*L1* and *CTLA4* expression (p = 0.01 and p = 0.03, respectively) (Fig. 3). To evaluate possible relationships between patients’ survival, miR-33a, and *PD*-*1*, we grouped lung adenocarcinoma patients according to the co-expression of both factors (miR-33a and *PD*-*1*). We then compared the difference in survival between the two groups linked to a negative association, high miR-33a/low *PD*-*1* and low miR-33a/high *PD*-*1* expression. We found that the first group of patients, with high miR-33a and low *PD*-*1*, showed a better prognosis, either for DFI or OS, than the opposite group (p = 0.04 and p = 0.007, respectively) (Fig. 4), implicating miR-33a as a good prognostic marker as a consequence of *PD*-*1* regulation.

### TCGA data analysis

A cohort of 323 lung adenocarcinoma patients from TCGA database was further analyzed, in order to validate our findings on a larger population. The samples were divided into high and low miR-33a expression groups based on the median value, and statistical analysis revealed that high miR-33a levels were significantly associated with low PD-1 expression (t test; p = 0.03), as we found by NanoString methodology. Moreover, survival analysis confirmed the association between miR-33a high expression and a better survival (p = 0.004 for DFI, and p = 0.007 for OS) (data not shown).

## Discussion

Gaining insight into the molecular basis of lung cancers is of critical clinical relevance in order to identify subgroups of patients for a more accurate management. Several studies have identified miRNA signatures with diagnostic and prognostic relevance [18, 19]. MiRNAs potentially target hundreds of different mRNAs, thus regulating a wide variety of cellular processes [20]. miR-33a has been shown to have potential tumor-suppressive activity by downregulating the expression of beta-catenin [5], but an improved deeper understanding of the role of this miRNA and its alternative antitumor mechanism is needed. In the current study, we showed that high miR-33a expression was associated with several favorable clinical–pathological characteristics, such as young age, female gender, low tumor grade, and early stage. Survival analysis also confirmed the good prognostic value of miR-33a. Our analysis next focused on the putative role of miR-33a in regulating the expression of immune markers in order to account for the antitumor function of miR-33a. The PD-1 receptor is a member of the immunoglobulin CD28 family, playing a crucial role in immune escape during tumor progression [21]. PD-L1 is expressed in different cancer types, including lung cancer, and its interaction with PD-1 plays an important role in the blockade of the “cancer immunity cycle” [22]. One of the most promising approaches in cancer, including lung adenocarcinoma, is antibody blockade of the PD-1/PDL-1 pathway [23–25]. This approach raises several questions, in particular whether PD-1/PD-L1 expression status is important in order to select patients eligible for these treatments, which technique is best suited in evaluating their levels, if these tumor immunity factors may be regulated by miRNAs, and how to define a threshold for positive PD-1/PD-L1 staining of tissue samples, considering that certain patients respond to treatment targeting PD-L1/PD-1, despite low or absent immunoreactivity for these biomarkers. Previously published studies suggest that tumor PD-L1 protein expression may be evaluated on human cancers using immunohistochemistry (IHC) in FFPE samples [6, 26]; however, several factors may impact the evaluation of PD-L1 positivity by conventional IHC, such as the specificity and reproducibility of the commercially available antibodies as well as the subjectivity of the staining interpretation. Velcheti et al. [27] reported a novel method of in situ measurement of PD-L1 mRNA NanoString nCounter technology, suggesting its utility for the accurate measurement of PD-1/PDL-1 levels in predicting the response of lung adenocarcinoma patients to targeted immunotherapy. NanoString employs unamplified nucleic acids and no cDNAs or enzymatic reaction step and is, therefore, less sensitive to tissue fixation effects; in our experience, this platform was extremely suitable to operate at very low levels of expression. Our findings provide a proof-of-principle that transcriptome-based analysis is a highly sensitive and effective means for PD-1/PD-L1 testing. Moreover, further analysis on a cohort of 323 lung adenocarcinoma patients from the TCGA database confirmed our findings on a larger population and also using a different transcriptome-based technology, such as Illumina HiSeq quantification.

The finding that targeting the PD-1/PD-L1 mechanism is of crucial importance in lung adenocarcinoma has led to increasing search for biomarkers predictive of response. Even if Nivolumab, an antibody targeting PD-1, has recently received Food and Drug Administration (FDA) approval for NSCLC therapy, regardless of the PDL1 expression status, a better prediction of which patients are more likely to respond to this cancer therapy may improve the treatment costs. Moreover, the effects of PD-1/PD-L1 signaling in the outcome of several neoplasms, including lung cancer, are not completely understood due to contradictory results [28, 29]. An alternative strategy in regulating immune response to tumors could be the expression of miRNAs that target immune checkpoint mRNAs. Using miRNA target prediction tools, we demonstrated a direct interaction of miR-33a and the 3′UTR of PD-1 and PD-L1 mRNAs, with consequent downregulation of the expression of both genes. In most circumstances, miRNAs bind to the target mRNA at the 3′ UTR region [30] and few of them were also reported to modulate genes through binding with the CDS region [31–33]. The target genes of miR-33a were surveyed globally by examining 3′UTR and CDS. Three putative binding sites of miR-33a-5p were found in the CDS region of the CTLA4 gene with similar miRanda scores.

Moreover, we identified a subset of lung cancer patients with high miR-33a levels and low PD-1 expression that had a favorable outcome, suggesting a better prognostic value of miR-33a via PD-1 regulation. Taken together, our findings identify a novel mechanism of tumor immune evasion regulated by miR-33a via PD-1/PD-L1, with potential application in clinical practice.

## Conclusion

Our data present novel insights into PD-1/PD-L1 signaling, first, regarding a novel reliable method for their evaluation, alternative to immunohistochemical testing, second, concerning miRNAs as modulators of immune checkpoint mechanisms, and finally regarding the potential value of miR-33a as a favorable prognostic marker via PD-1 regulation. MiRNA analysis combined with tumor expression of immune-biomarkers will improve our ability to select the best candidates to receive immune-based therapies, with important clinical benefits.
